# Commentary: Anti-TNFα in inflammatory bowel disease: from originators to biosimilars

**DOI:** 10.3389/fphar.2025.1625961

**Published:** 2025-07-07

**Authors:** Yingjie Liu, Keying Wang, Wenjiang Wu

**Affiliations:** Shenzhen Hospital (Futian) of Guangzhou University of Chinese Medicine, Shenzhen, China

**Keywords:** IBS, inflammatory bowel disease, anti-TNFα, originators, biosimilars

## Introduction

The review by Zeng et al. (2024) provides a timely synthesis of clinical and economic implications of TNFα biosimilars in inflammatory bowel disease (IBD). While the article addresses critical aspects of biosimilar adoption, several methodological and contextual limitations warrant clarification to enhance its academic rigor and relevance. This commentary highlights key concerns, proposes actionable revisions, and underscores the importance of addressing these gaps to solidify the article’s utility for clinicians and policymakers.

## Literature currency and scope

### Outdated references

The review cites cost-saving data from 2017 U.S. biologics expenditures (Kappelman et al., 2008), which fails to reflect recent market dynamics. Furthermore, the review’s overall assessment of the evidence landscape appears inadequately updated. Consider the systematic review by Martelli and Peyrin-Biroulet (2019), which synthesized the state of knowledge *up to 2016*. Martelli et al. concluded that while infliximab biosimilars showed promise in IBD, significant knowledge gaps regarding long-term safety, interchangeability, and real-world effectiveness in complex populations persisted, necessitating large post-marketing studies. A 2024 review has the responsibility to critically evaluate how these gaps identified in 2019 have been addressed by subsequent evidence (e.g., Schreiber et al., 2021; Kennedy et al., 2019 on long-term safety; NOR-SWITCH extension on interchangeability; IQVIA 2023 on market penetration and cost savings). Failing to incorporate and discuss these key post-2019 developments - particularly immunogenicity monitoring strategies validated in prospective cohorts (Kennedy et al., 2019) - significantly diminishes the review’s currency and utility for understanding the current biosimilar landscape in IBD.

### Evidence imbalance

The authors heavily rely on rheumatoid arthritis (RA) studies (e.g., VOLTAIRE-CD) to support biosimilar efficacy, while IBD-specific long-term safety data remain underrepresented. For instance, CT-P13’s safety profile in IBD cohorts beyond 2 years is scarcely discussed, despite robust evidence demonstrating the impact of immunogenicity on long-term outcomes (Schreiber et al., 2021; Kennedy et al., 2019). Prioritizing IBD-centric studies would align the review’s scope with its clinical focus.

## Data consistency and methodological gaps

### Contradictory claims

The manuscript states both “low biosimilar uptake in the U.S.” and “substantial cost savings,” creating ambiguity. Clarifying temporal trends—such as the 44% U.S. infliximab biosimilar market penetration by 2023 (IQVIA Institute, 2023)—would reconcile these statements. Additionally, regional disparities in biosimilar adoption (e.g., Europe’s 88% uptake vs the U.S.’s delayed growth) merit exploration to avoid oversimplification.

### Small sample bias

Conclusions drawn from underpowered studies (e.g., PF-06438179/GP1111, *n* = 10) lack statistical robustness. This concern regarding methodological rigor and statistical power in IBD biosimilar research is not new. As acknowledged in the Martelli and Peyrin-Biroulet (2019) systematic review, much of the *early real-world evidence* supporting biosimilars in IBD, upon which subsequent understanding was built, stemmed from observational studies with inherent limitations in size and design. Martelli et al.'s call for ‘large prospective post-marketing studies’ underscores the recognized need for more robust data. Therefore, Zeng et al.’s inclusion and interpretation of conclusions from very small studies like PF-06438179/GP1111, without explicitly emphasizing their preliminary nature and placing them in the context of this established need for larger trials (such as the NOR-SWITCH extension study, *n* = 380; Goll et al., 2019), weakens the review’s evidence synthesis. Highlighting confidence intervals and the limitations of small samples is crucial to avoid overinterpreting findings.

## Underdeveloped discussions

### Immunogenicity oversight

While the review acknowledges biosimilar immunogenicity, its clinical implications—such as antibody-guided dosing (Strand et al., 2020) or the impact of multiple switches—are underexplored. Martelli and Peyrin-Biroulet (2019) specifically identified immunogenicity as a key area requiring deeper understanding, noting that while early data suggested a ‘similar immunogenicity profile’ for CT-P13 compared to the originator, the clinical consequences of immunogenicity in real-world settings and during switching scenarios were still being actively investigated (e.g., the then-ongoing NOR-SWITCH trial). Given that immunogenicity directly impacts treatment efficacy - increasing failure risk by 3.1-fold as shown in IBD cohorts (Kennedy et al., 2019) - a more nuanced discussion in Zeng et al.'s review was warranted. They could have built upon the foundation laid by Martelli et al. by incorporating subsequent findings on immunogenicity monitoring strategies (e.g., trough levels, antibody testing), the implications for switching decisions, and data exploring potential differences arising from multiple switches, which remain critical concerns for clinicians managing IBD.

### Policy solutions

The manuscript omits actionable strategies to overcome patent barriers and reimbursement challenges. Lessons from the EU’s compulsory licensing framework or the FDA’s interchangeable designation for BI 695501 (adalimumab biosimilar) could provide policymakers with tangible pathways to enhance biosimilar accessibility.

## Terminology and presentation issues

### Semantic precision

The conflation of “biosimilar” and “generic” in Section *Contradictory claims* misrepresents distinct regulatory pathways. Biosimilars require comparative clinical trials, whereas generics rely solely on bioequivalence. Correcting this misclassification is essential to avoid confusion.

### Figure incompleteness

The TNFα signaling pathway described by Zeng et al. (2024) (Figure 1), requires primary source attribution, reducing reproducibility. The biosimilar approval process should align with World Health Organization (2022), which would improve clarity, particularly in delineating the abbreviated approval pathway for biosimilars versus originators.

## Additional recommendations

### Structural flow

Transitions between Sections *Underdeveloped discussions* (benefits) and *Terminology and presentation issues* (challenges) are abrupt. Integrating cost-saving data with discussions on policy barriers (e.g., rebate traps in the U.S.) would enhance narrative cohesion.

### Future directions

The review overlooks precision medicine advancements, such as biomarkers for biosimilar switching (e.g., trough drug levels, fecal calprotectin). Incorporating these aligns the manuscript with the “treat-to-target” paradigm and addresses personalized therapeutic monitoring.

## Discussion

Zeng et al.’s review lays a foundational understanding of anti-TNFα biosimilars in IBD but requires revisions to achieve its potential as a pivotal resource. Updating literature, harmonizing data, and deepening critical analysis—particularly regarding immunogenicity, policy, and precision medicine—are imperative. Addressing these concerns will not only bolster academic rigor but also empower clinicians and policymakers to navigate the evolving biosimilar landscape confidently.

Future iterations should emphasize real-world evidence, regional adoption disparities, and innovative regulatory strategies to reflect the dynamic interplay between biosimilar accessibility and healthcare sustainability. By doing so, the review can serve as a comprehensive guide for optimizing IBD management in an era of biologic competition.
